# Expression of Lysyl Oxidase-Related Protein and Effect of Lysyl Oxidase Inhibition in Cyclosporine-Induced Nephropathy Mouse Model

**DOI:** 10.3390/ph19060960

**Published:** 2026-06-21

**Authors:** Hyo Jeong Kim, Tae Yeon Kim, Jong Hyun Jhee, Hoon Young Choi, Jae Myun Lee, Hyeong Cheon Park

**Affiliations:** 1 Department of Internal Medicine, National Health Insurance Service Medical Center, Ilsan Hospital, 100 Ilsan-ro, Goyang 10444, Republic of Korea; tojeong613@gmail.com; 2 Division of Nephrology, Department of Internal Medicine, Gangnam Severance Hospital, Yonsei University College of Medicine, 211 Eonju-ro, Gangnam-gu, Seoul 06273, Republic of Korea; tyeon_81@naver.com (T.Y.K.); jjhlove77@yuhs.ac (J.H.J.); hychoidr@yuhs.ac (H.Y.C.); 3 Severance Institute for Vascular and Metabolic Research, Yonsei University College of Medicine, Seoul 03722, Republic of Korea; 4 Department of Microbiology and Immunology, Yonsei University College of Medicine, 50-1 Yonsei-ro, Seodaemun-gu, Seoul 03722, Republic of Korea

**Keywords:** chronic kidney disease, cyclosporine, kidney fibrosis, lysyl oxidase-like 2

## Abstract

**Background/Objectives** Lysyl oxidase-like 2 (LOXL2), a member of the lysyl oxidase family of amine oxidases involved in collagen cross-linking, has emerged as a key mediator of pathological extracellular matrix remodeling and tissue fibrosis. Dysregulated LOXL2 activity has been implicated in various fibrotic diseases; however, its role in fibrosis-driven chronic kidney injury, particularly in the context of calcineurin inhibitor-induced kidney toxicity, remains incompletely defined. **Methods** To investigate the contribution of LOXL2 inhibitor to cyclosporine A (CsA)-induced nephropathy, a well-established model of progressive tubulointerstitial fibrosis, male CD-1 mice were administered either saline or CsA (15 mg/kg/day, intraperitoneally) for 8 weeks. After 4 weeks of CsA exposure, CsA-treated mice were further divided into two groups and received either vehicle or a LOXL2 inhibitor (10 mg/kg/day, oral gavage) for an additional 4 weeks. Kidney function, albuminuria, histological fibrosis, inflammatory cell infiltration, and profibrotic gene expression were assessed. **Results** In a murine model of CsA-induced nephropathy, pharmacological inhibition of LOXL2 markedly improved kidney outcomes. LOXL2 inhibition significantly reduced albuminuria and ameliorated kidney dysfunction. In parallel, tubulointerstitial fibrosis was substantially attenuated, accompanied by reduced myofibroblast activation and extracellular matrix accumulation. These protective effects were associated with downregulation of profibrotic and inflammatory mediators and inhibition of TGF-β-related downstream signaling pathways activated by CsA. **Conclusions** The present preclinical findings suggest that Compound #765-mediated LOXL2 inhibition may offer a potential therapeutic benefit in CsA-induced fibrosis, though further validation is warranted.

## 1. Introduction

Long-term immunosuppressive therapy is essential for kidney transplantation and managing immune-mediated diseases; however, chronic nephrotoxicity remains a major limitation. Calcineurin inhibitors, including cyclosporine A (CsA), are widely used immunosuppressive agents, and their long-term use is associated with progressive kidney dysfunction that substantially compromises patient and graft outcomes [1,2].

Chronic nephrotoxicity induced by calcineurin inhibitors is characterized by progressive tubulointerstitial fibrosis, which represents a key pathological determinant of irreversible kidney dysfunction [3]. Experimental and clinical studies have demonstrated that excessive accumulation and stabilization of extracellular matrix (ECM) components occur early after calcineurin inhibitor exposure and precede functional decline [2]. This fibrosis-dominant phenotype has been consistently observed in kidney transplant recipients receiving long-term calcineurin inhibitor therapy, as well as in experimental models of calcineurin inhibitor-induced nephropathy [4]. Despite extensive investigation of upstream profibrotic signaling pathways, therapeutic strategies directly targeting ECM remodeling in calcineurin inhibitor-associated kidney fibrosis remain limited.

The lysyl oxidase (LOX) family comprises copper-dependent amine oxidases that catalyze the crosslinking of collagen and elastin in the ECM, thereby promoting matrix stabilization and increasing tissue stiffness [5,6]. Accumulating evidence indicates that LOX expression is upregulated in various fibrotic conditions and is closely associated with profibrotic signaling pathways such as transforming growth factor-β (TGF-β) [7]. In experimental models, increased LOX activity has been linked to enhanced ECM deposition, fibroblast activation, and tissue fibrosis progression [7]. Importantly, pharmacological inhibition of LOX has been shown to attenuate fibrosis in multiple organ systems, suggesting that targeting ECM crosslinking represents a promising therapeutic strategy [8,9,10]. Among the LOX family members, lysyl oxidase-like 2 (LOXL2) has been particularly implicated in fibrotic remodeling by mediating the cross-linking of type IV collagen, a key structural component of the glomerular basement membrane [7]. However, the role of LOXL2 inhibitor in calcineurin inhibitor-induced nephropathy remains poorly defined. Thus, we explored the role of LOXL2 in CsA-induced kidney fibrosis and assessed the therapeutic potential of LOXL2 inhibitors in mitigating this fibrotic process. To this end, we employed Compound #765, a LOXL2 inhibitor developed in our previous study, as a pharmacological tool to evaluate its efficacy in a CsA-induced kidney fibrosis model.

## 2. Results

### 2.1. Clinical Characteristics and Kidney Function Changes After CsA and LOXL2 Inhibitor Treatment

**Figure 1:**
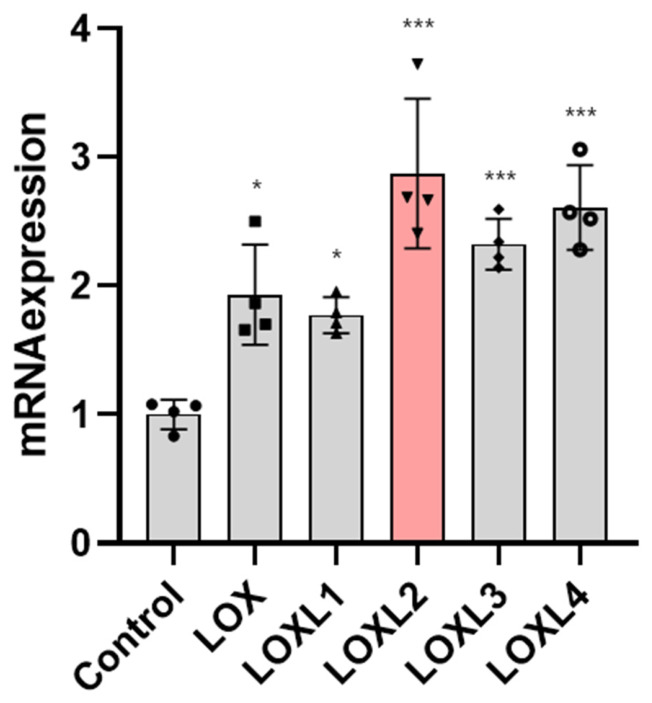
LOX family expression changes after CsA administration. Data are expressed as mean ± SD vs. control (n = 4 in each experiment group). Statistical comparisons were performed using one-way ANOVA with Turkey’s post hoc test vs. control: * *p* < 0.05, *** *p* < 0.001. Abbreviation: CsA, cyclosporin A; LOX, lysyl oxidase; LOXL, lysyl oxidase-like; SD, standard deviation.

Before proceeding to the main experiment, the mRNA gene expression of the LOX and LOXL families was compared in CsA-induced mice and found to significantly increase after CsA administration (LOX: *p* = 0.010; LOXL1: *p* = 0.042; LOXL2: *p* < 0.001; LOXL3: *p* < 0.001; LOXL4: *p* < 0.001, Figure 1, Supplementary Table S1). Typically, among these LOX and LOXL families, a notable upregulation was observed in LOXL2, which showed a 2.87-fold increase compared to the control (mean difference = −1.87, 95% CI: −2.62 to −1.12, *p* < 0.001). Based on this finding, LOXL2 and its inhibitor were studied to examine their role in kidney fibrosis in CsA-induced mice.

**Figure 2:**
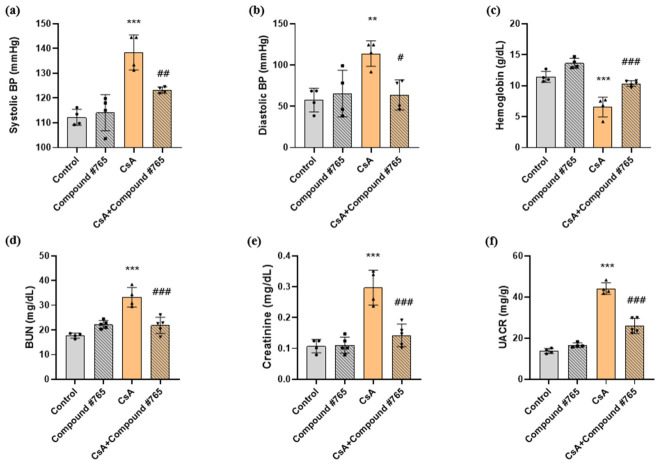
Clinical characteristics changes after CsA and Compound #765 administration. (**a**) systolic BP; (**b**) diastolic BP; (**c**) Hb; (**d**) BUN; (**e**) Creatinine; (**f**) UACR. Data are expressed as mean ± SD (n = 4–5 in each experiment group). Statistical comparisons were performed using one-way ANOVA with Tukey’s post hoc test vs. control: ** *p* < 0.01, *** *p* < 0.001; vs. CsA: # *p* < 0.05, ## *p* < 0.01, ### *p* < 0.001. Abbreviation: BP, blood pressure; BUN, blood level of urea nitrogen; CsA, cyclosporin A; Hb, hemoglobin; SD, standard deviation; UACR, urine albumin-to-creatinine ratio.

Significant changes in blood pressure were observed after CsA administration, as well as in laboratory parameters, reflecting kidney dysfunction (Figure 2a–f, Supplementary Table S2). Compared with control mice, CsA-induced mice showed significantly higher blood urea nitrogen (BUN; 17.73 ± 0.88 vs. 33.25 ± 3.45 mg/dL, *p* < 0.001) and creatinine levels (0.11 ± 0.02 vs. 0.30 ± 0.05 mg/dL, *p* < 0.001). After Compound #765 treatment, there was an improvement in kidney function, showing a significant decrease in BUN (33.25 ± 3.45 vs. 21.84 ± 2.95 mg/dL, *p* < 0.001), and creatinine levels (0.30 ± 0.05 vs. 0.14 ± 0.03 mg/dL, *p* < 0.001) compared to CsA-treated mice. Similarly, the urine albumin-to-creatinine ratio (UACR) significantly increased following CsA administration (13.78 ± 1.06 vs. 44.15 ± 2.46 mg/g, *p* < 0.001), and UACR significantly decreased after Compound #765 treatment (44.15 ± 2.46 vs. 25.98 ± 3.25 mg/g, *p* < 0.001). Furthermore, hemoglobin level significantly decreased after CsA administration, and significant improvement was observed in CsA + Compound #765-treated mice (Figure 2c).

### 2.2. Histopathological Changes in CsA-Induced Mice and LOXL2-Inhibitor-Treated Mice

**Figure 3:**
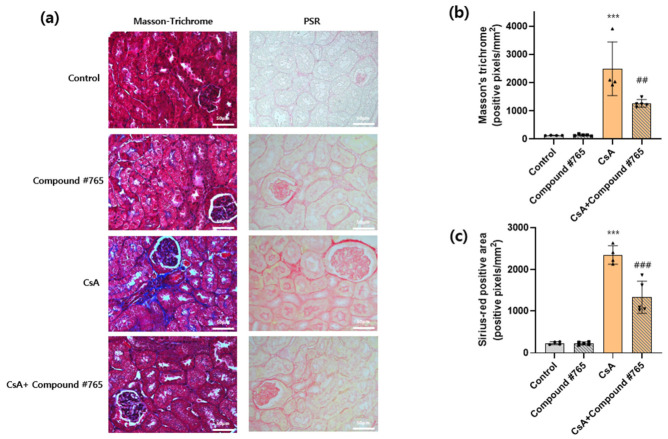
Histopathological changes after CsA and Compound #765 administration. (**a**) Images of histological staining; (**b**) Masson-Trichrome staining; (**c**) PSR staining. Data are expressed as mean ± SD (n = 4–5 in each experiment group). Statistical comparisons were performed using one-way ANOVA with Tukey’s post hoc test vs. control: *** *p* < 0.001; vs. CsA: ## *p* < 0.01, ### *p* < 0.001. Abbreviation: CsA, cyclosporin A; PSR. picrosirius red; SD, standard deviation.

In line with laboratory tests, CsA administration also induced histopathological changes. Masson’s trichrome (MT) staining was used to assess the extent of fibrosis, and Picrosirius Red (PSR) staining was further performed to specifically highlight collagen fibers, particularly type I and III collagen. As seen in Figure 3a, interstitial inflammation and fibrosis increased in CsA-induced mice, with a significant elevation in MT staining-positive area. A similar pattern was observed in PSR staining, emphasizing the development of fibrotic changes within the kidney interstitium. This outcome correlates with a significant decrease in kidney function studied in laboratory tests. Furthermore, in the group of mice treated with Compound #765 following CsA induction, a statistically significant decrease in MT and PSR staining was observed compared to CsA-induced mice (Figure 3b,c, Supplementary Table S3). These results support the effectiveness of Compound #765 in ameliorating kidney fibrosis within the tubulointerstitium.

### 2.3. Suppression of ECM Proteins in LOXL2 Inhibitor-Treated Mice

**Figure 4:**
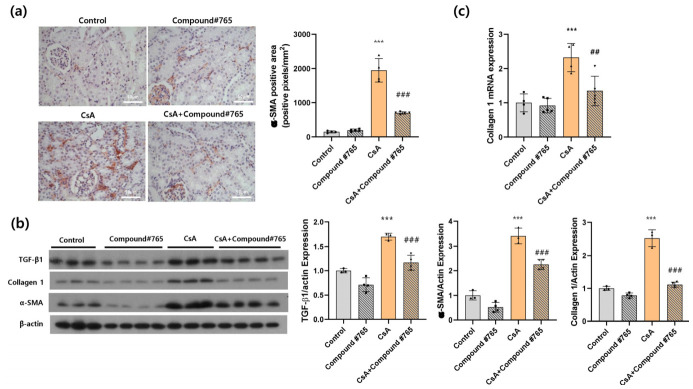
Changes in ECM proteins after CsA and Compound #765 administration. (**a**) Immunohistochemistry of α-SMA; (**b**) Western blots of TGF-β1, collagen type 1A and α-SMA; (**c**) mRNA expression of collagen type 1A. TGF-β1, collagen type 1A, and α-SMA were probed sequentially on the same membrane, with β-actin used as a single shared loading control following membrane reprobing. Data are expressed as mean ± SD (n = 4–5 in each experiment group). Statistical comparisons were performed using one-way ANOVA with Tukey’s post hoc test vs. control: *** *p* < 0.001; vs. CsA: ## *p* < 0.01, ### *p* < 0.001. Abbreviation: α-SMA, α-smooth muscle actin; CsA, cyclosporin A; ECM, extracellular matrix; SD, standard deviation; TGF, transforming growth factor.

Alpha-smooth muscle actin (α-SMA) is a protein associated with kidney fibrosis and is expressed in myofibroblasts, which is a major source of ECM proteins that contributes to the development and progression of kidney tubulointerstitial fibrosis. Immunohistochemical staining demonstrated an increase in α-SMA expression in CsA-induced mice. By contrast, the group administered with Compound #765 showed a significant reduction in the α-SMA positive area, indicating an improvement in kidney fibrosis (*p* < 0.001, Figure 4a, Supplementary Table S4). Western blot analysis further revealed that TGF-β1, α-SMA and collagen protein expressions were markedly upregulated following CsA administration and significantly decreased after Compound #765 treatment (*p* < 0.001, Figure 4b). Of note, these three proteins were assessed on the same membrane via sequential reprobing, with β-actin serving as the common loading control. Additionally, reverse transcription quantitative polymerase chain reaction (RT-qPCR) analysis showed an increase in collagen mRNA expression following CsA treatment, with a subsequent decrease in collagen mRNA expression observed after Compound #765 treatment (*p* = 0.004, Figure 4c).

**Figure 5:**
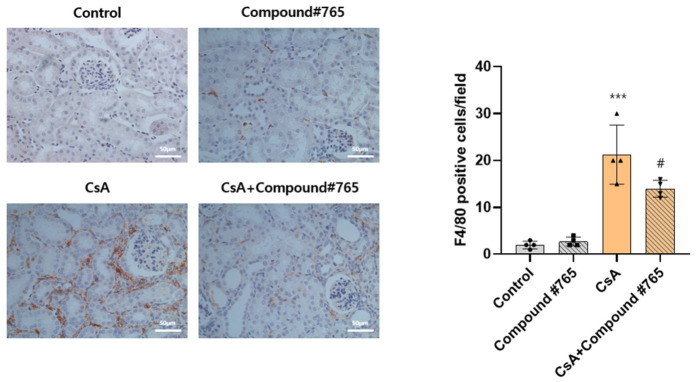
Changes in immunohistochemical staining of F4/80 after CsA and Compound #765 administration. Data are expressed as mean ± SD (n = 4–5 in each experiment group). Statistical comparisons were performed using one-way ANOVA with Tukey’s post hoc test vs. control: *** *p* < 0.001; vs. CsA: # *p* < 0.05. Abbreviation: CsA, cyclosporin A; SD, standard deviation.

**Figure 6:**
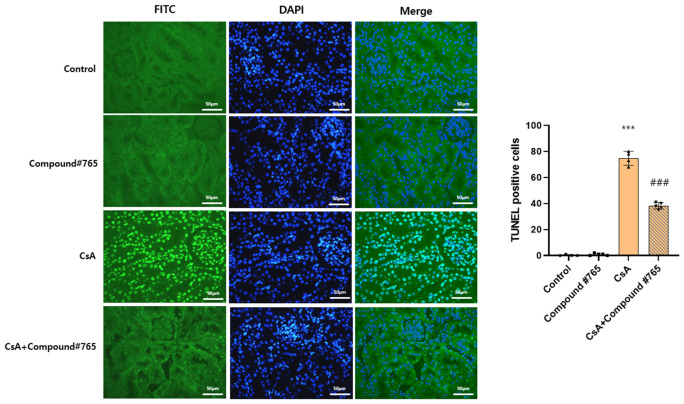
Changes in immunohistochemical staining of TUNEL after CsA and Compound #765 administration. Data are expressed as mean ± SD (n = 4–5 in each experiment group). Statistical comparisons were performed using one-way ANOVA with Tukey’s post hoc test vs. control: *** *p* < 0.001; vs. CsA: ### *p* < 0.001. Abbreviation: CsA, cyclosporin A; DAPI, 4′,6-Diamidino-2-phenylindole; FITC, fluorescein isothiocyanate; SD, standard deviation; TUNEL, terminal deoxynucleotidyl transferase dUTP nick end labeling.

In addition, immunohistochemical staining of F4/80 and transferase dUTP nick end labeling (TUNEL) were performed to further determine the effect of Compound #765 treatment. The number of F4/80-positive inflammatory cells increased after CsA administration, but the infiltrations were significantly suppressed by Compound #765 treatment (*p* < 0.05, Figure 5, Supplementary Table S5). Consistent with the observed increase in F4/80-positive inflammatory cells after CsA administration, we also noted a corresponding rise in TUNEL-positive cells (*p* < 0.001, Figure 6, Supplementary Table S6). However, notably, Compound #765 treatment significantly attenuated this apoptotic response, as evidenced by a decrease in the number of TUNEL-positive cells (*p* < 0.001, Figure 6), which indicates a potential protective effect of Compound #765 against apoptosis induced by CsA administration.

### 2.4. Suppression of the Expression of Pro-Inflammatory Cytokines and Downstream Signaling Pathways by LOXL2 Inhibition

**Figure 7:**
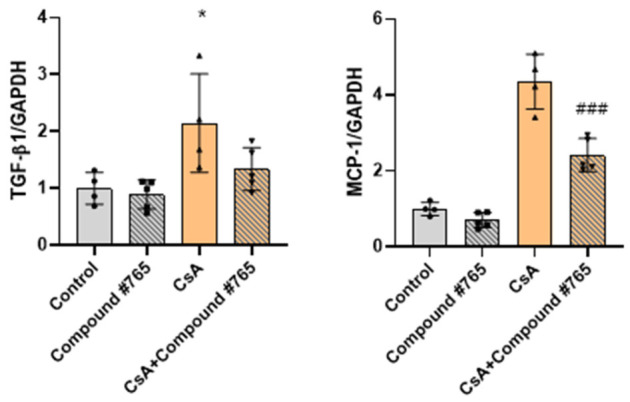
Changes in proinflammatory cytokines after CsA and Compound #765 administration. Data are expressed as mean ± SD (n = 4–5 in each experiment group). Statistical comparisons were performed using one-way ANOVA with Tukey’s post hoc test vs. control: * *p* < 0.05; vs. CsA: ### *p* < 0.001. Abbreviation: CsA, cyclosporin A; GAPDH, glyceraldehyde-3-phosphate dehydrogenase; MCP, monocyte chemoattractant protein; SD, standard deviation; TGF, transforming growth factor.

Quantitative RT-PCR and Western blot analyses showed that TGF-β1 gene and protein expression were significantly increased in CsA-induced mice. While no significant decrease was observed in the CsA + Compound #765-treated group in RT-qPCR, a significant reduction was evident in Western blot following Compound #765 administration (*p* < 0.001 Figure 4b and Figure 7, Supplementary Tables S4 and S7). These results indicate that CsA administration leads to an increase in pro-inflammatory cytokines, which is subsequently attenuated following Compound #765 treatment. Additionally, the elevated monocyte chemoattractant protein (MCP)-1 gene expression observed in CsA-induced mice was significantly reduced after Compound #765 administration (*p* < 0.001, Figure 7). These results collectively support the therapeutic effect of Compound #765 in suppressing CsA-induced inflammation.

**Figure 8:**
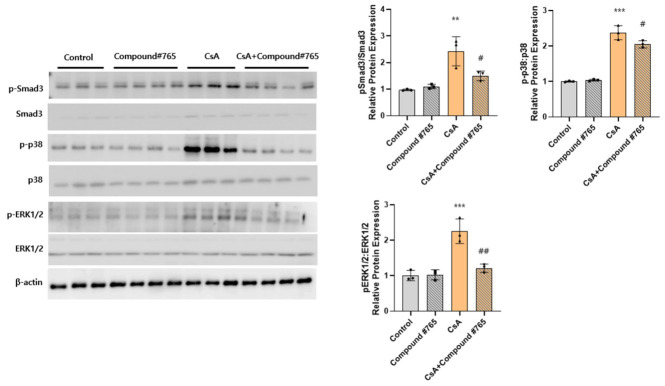
Changes in Smad3 and MAPK signaling activation after CsA and Compound #765 administration. Data are expressed as mean ± SD (n = 4–5 in each experiment group). Statistical comparisons were performed using one-way ANOVA with Tukey’s post hoc test vs. control: ** *p* < 0.01, *** *p* < 0.001; vs. CsA: # *p* < 0.05, ## *p* < 0.01. Abbreviation: CsA, cyclosporin A; MAPK, mitogen-activated protein kinase; SD, standard deviation.

Consistent with these findings, Western blot analyses further demonstrated that CsA administration markedly activated key inflammatory and profibrotic signaling pathways. Specifically, CsA treatment significantly increased the phosphorylated p38 (p-p38)/p38, phosphorylated Smad3 (p-Smad3)/Smad3, and phosphorylated ERK1/2 (p-ERK1/2)/ERK1/2 ratios. By contrast, Compound #765 treatment markedly attenuated these increases, indicating suppression of downstream mitogen-activated protein kinase and TGF-β/Smad signaling pathways (Figure 8, Supplementary Table S8).

### 2.5. LOXL2 Inhibition Attenuates CsA-Induced Fibrosis in HK-2 Cells

To further validate the mechanistic basis of the antifibrotic effects, we performed additional experiments using HK-2 human proximal tubular epithelial cells. Prior to the fibrosis experiment, cell viability was assessed across a range of Compound #765 concentrations (0.5, 1.0, 2.0, and 5.0 µM) in the presence of CsA. Cell viability remained above 90% at concentrations up to 1.0 µM, which was therefore selected as the working concentration for subsequent experiments (Supplementary Figure S1).

HK-2 cells were treated with CsA (5 µM) in the presence or absence of Compound #765 (1 µM) for 24 h. CsA treatment significantly upregulated the mRNA expression of TGF-β1, Collagen type 1A, and α-SMA compared to control cells, whereas co-treatment with Compound #765 significantly attenuated the CsA-induced upregulation of all three fibrosis-related markers (Supplementary Table S9 and Figure S2). These findings demonstrate that Compound #765 directly suppresses fibrotic gene expression in tubular epithelial cells, providing mechanistic support for the antifibrotic effects observed in our murine model.

## 3. Discussion

The present study was designed to investigate the contribution of LOXL2 to CsA-induced kidney fibrosis and assess the therapeutic potential of LOXL2 inhibitors. To evaluate the antifibrotic effects of a LOXL2 inhibitor, CsA-induced tubulointerstitial fibrosis model was established in CD-1 mice. CsA administration was found to significantly induce kidney dysfunction, as evidenced by increased levels of BUN, creatinine, and proteinuria, along with histopathological signs of interstitial inflammation and fibrosis. Notably, LOXL2 inhibitor (Compound #765) treatment ameliorated these fibrotic changes, reducing inflammation, apoptosis, and pro-inflammatory cytokine expression, indicating a protective effect against CsA-induced kidney fibrosis.

CsA, a calcineurin inhibitor widely used for immunosuppression in kidney transplantation, has paradoxically been associated with nephrotoxicity and fibrosis. CsA induces acute vasoconstriction of afferent arterioles, reducing kidney blood flow and leading to hypoxic injury and ischemia [1]. At the molecular level, CsA induces excessive generation of reactive oxygen species, resulting in oxidative tissue damage [11,12,13,14]. CsA also triggers inflammatory pathways, marked by immune cell infiltration and activation of pro-inflammatory cytokines like tumor necrosis factor-α, interleukin-6, and TGF-β, exacerbating kidney injury and fibrosis [15]. Chronic CsA exposure activates fibroblasts and myofibroblasts, resulting in excessive ECM deposition and tubulointerstitial fibrosis [1]. Therefore, new strategies to mitigate these adverse effects in CsA-induced nephrotoxicity are needed.

LOX and LOXL family members are established mediators of ECM stiffening through the catalytic cross-linking of fibrillar collagen [5,16,17]. Recent studies have focused on LOXL2 in its involvement in various fibrotic conditions, including liver cirrhosis [9,18], pulmonary fibrosis [19,20], and heart failure [21], where it functions as a crucial driver of pathological matrix remodeling. Although research on kidney fibrosis is still limited, accumulating evidence indicates that LOXL2 is disproportionately elevated in kidney failure patients relative to other LOX family members. Previous studies have demonstrated that hypoxia-inducible factor-responsive genes are upregulated in kidney tissue from patients with chronic kidney disease [22]. Notably, LOXL2 emerged as the most consistently overexpressed gene across distinct nephropathy subtypes, encompassing diabetic nephropathy, IgA nephropathy and hypertensive nephrosclerosis, thereby implicating this enzyme as a key contributor to fibrotic disease development in the kidney [22,23]. Similarly, in our study on CsA-induced mice, we found that LOXL2 exhibited the highest expression among the LOX and LOXL family members. This finding supports previous studies suggesting that LOXL2 might be an important target in kidney fibrosis treatment.

Despite the well-known effects of LOXL2 on fibrosis, the therapeutic effects of LOXL2 inhibition have shown variable results across studies. In experimental models, LOXL2 inhibition in Alport mice was shown to suppress the expression of inflammatory cytokines and proteins. This effect led to reduced glomerulosclerosis and interstitial fibrosis, ultimately preserving kidney function [24]. Conversely, another study on a diabetic nephropathy model demonstrated more limited effects, with improvement confined to glomerular fibrosis, and minimal impact on the tubulointerstitium [25]. These discrepancies may be attributable to differences in the predominant site of injury, the stage of fibrosis at the time of treatment, and the underlying pathological mechanism of each disease model. In CsA-induced nephropathy, tubulointerstitial fibrosis is the hallmark pathological feature, and LOXL2 was found to be the most highly upregulated member of the LOX family in our model (Figure 1), providing a strong rationale for targeting LOXL2 specifically in this context. Moreover, other studies have raised questions about the necessity of targeting LOXL2 alone and have suggested that dual inhibitors are more effective in preventing fibrosis [26]. While dual inhibition may offer broader antifibrotic coverage, our results suggest that selective LOXL2 inhibition is sufficient in attenuating tubulointerstitial fibrosis in the CsA nephropathy model, likely due to the predominant role of LOXL2 in this specific pathological setting. Nonetheless, given that LOX family enzymes are indispensable for sustaining normal ECM homeostasis, non-specific or excessive inhibition may lead to unintended adverse effects. Given these considerations, our study focused on evaluating the therapeutic effects of a LOXL2-specific inhibitor in a CsA-induced nephropathy model. Our findings demonstrate that LOXL2 inhibition not only attenuated established fibrosis but also suppressed inflammation-related cytokines, thereby limiting the progression of kidney fibrosis and preventing further fibrotic remodeling.

This study has several limitations that warrant consideration. First, our findings are based on a murine model, and the translational relevance to human CsA-induced nephropathy remains to be established. Second, the relatively small sample size in each experimental group (n = 4–5) may limit the statistical power of our findings. Third, although we demonstrated that Compound #765 attenuated fibrosis-related gene expression both in vivo and in vitro, the precise upstream mechanisms by which LOXL2 inhibition modulates TGF-β and mitogen-activated protein kinase signaling pathways were not fully elucidated. Fourth, the selectivity of Compound #765 for LOXL2 over other LOX family members has not been fully characterized, and off-target effects on related isoforms at higher concentrations cannot be excluded. Furthermore, comprehensive preclinical toxicity and safety evaluations will be necessary prior to any translational application.

In conclusion, the present study demonstrates that LOXL2 plays a significant role in CsA-induced kidney fibrosis, and targeting LOXL2 with a specific inhibitor (Compound #765) effectively mitigates fibrosis and inflammatory responses. Collectively, these results support LOXL2 inhibition as a viable therapeutic strategy for mitigating CsA-induced nephrotoxicity and fibrotic progression.

## 4. Materials and Methods

### 4.1. Drugs and Chemicals

Compound #765 (6-Amino-5-(4-methoxybenzoyl)indolizine-7-carbonitrile; molecular formula C_17_H_13_N_3_O_2_; molecular weight 291.30 g/mol) is a novel indolizine derivative synthesized, characterized, and reported in our previous study [27]. Its chemical identity and purity were confirmed using ^1^H/^13^C nuclear magnetic resonance spectroscopy (AVANCE III HD 600 MHz, Bruker, Billerica, MA, USA), high-resolution mass spectrometry (Xevo G2-XS QTof, Waters, Milford, MA, USA), and high-performance liquid chromatography (Agilent 1260 Infinity II, Agilent Technologies, Santa Clara, CA, USA; purity of 99.4% at λ = 254 nm). A previous study demonstrated that Compound #765 potently inhibited LOXL2 enzymatic deaminase activity in a dose-dependent manner. In addition, direct interaction with LOXL2 was supported by a Drug Affinity Responsive Target Stability assay and in silico molecular docking analysis, which predicted preferential binding to the catalytic groove of LOXL2. Compound #765 is protected under Korean Patent No. 10-2631074 (filed 9 April 2021; registered 25 January 2024), assigned to the Industry-Academic Cooperation Foundation of Yonsei University, Seoul, Republic of Korea. Stock solutions of Compound #765 were prepared in dimethyl sulfoxide (DMSO) and stored at −20 °C. Working solutions were freshly prepared before each administration, and the final DMSO concentration in all assays did not exceed 0.1% (*v*/*v*).

### 4.2. Animal Model

A total 18 male CD-1 mice (6–8 weeks old; Orient Bio, Seongnam, Republic of Korea) were housed under pathogen-free conditions in a facility maintained at 20–22 °C with a 12-h light/dark cycle, with free access to water and food. The animals were divided into four groups: olive oil group (control), olive oil+ Compound #765 group, CsA group, and CsA+ Compound #765 group. Tubulointerstitial fibrosis was induced in nine mice by daily intraperitoneal injection of CsA (15 mg/kg/day, Chong Kun dang pharmaceutical Corp, Seoul, Republic of Korea) for eight weeks. The mice were divided into two groups after four weeks of CsA administration; one group continued to receive only CsA, while the other received an additional Compound #765 (10 mg/kg/day, provided by Department of Microbiology, Yonsei University College of Medicine, Seoul, Republic of Korea) administered by oral gavage. Control male CD-1 mice (n = 9) were injected intraperitoneally with olive oil daily (1 mg/kg/day, Sigma-Aldrich, St. Louis, MO, USA) and were further divided into two groups after four weeks of olive oil administration: one group received only olive oil injections, while the other group received an additional administration of Compound #765.

Blood pressure was measured at the end of the eight-week experiment using a computerized non-invasive tail cuff system (BP-2000 Blood Pressure Analysis System; Visitech Systems, NC, USA). To minimize induced stress, the animals were accustomed to the instrument for four consecutive days prior to definitive measurements, with a minimum of 10 repeated measurements per mouse. Furthermore, mice were sacrificed, and blood samples were obtained by cardiac puncture. Subsequently, half of the kidneys were stored in liquid nitrogen, and another half were fixed in 4% formaldehyde for 24–48 h. The animal study protocol was designed in accordance with the guidelines for use of laboratory animals and approved by the Institutional Animal Care and Use Committee of Yonsei University Healthcare System (Approval number: IACUC, no. 2022-0006).

### 4.3. In Vitro Experiments Using HK-2 Cells

Human proximal tubular epithelial cells (HK-2; ATCC, Manassas, VA, USA) were cultured in DMEM/F-12 (Gibco, Grand Island, NY, USA) supplemented with 10% fetal bovine serum (FBS) and 1% penicillin/streptomycin at 37 °C in a 5% CO_2_ incubator. HK-2 cells were divided into four groups: (1) control, (2) Compound #765 alone (1 µM), (3) CsA alone (5 µM), and (4) CsA + Compound #765 and incubated for 24 h under the indicated treatment conditions.

### 4.4. Biomarker Assays of Kidney Function and Histology

Kidney function was evaluated using BUN and serum creatinine, and their concentrations were determined using a Fuji Dri-Chem 4000i system (Fujifilm, Japan). The urinary albumin concentration was measured using an ELISA kit (catalog no. ab-108792, Abcam, Cambridge, MA, USA), while the urinary creatinine concentration was measured using a creatinine colorimetric kit (catalog no. 500701, Cayman Chemical, Ann Arbor, MI, USA). The UACR was calculated as the urinary albumin concentration divided by the urinary creatinine concentration.

Harvested kidneys were embedded in paraffin, cut into 4 μm slices and stained with MT, and PSR. Interstitial fibrosis, inflammation, and collagen deposition were assessed and expressed as positive pixels per mm^2^ through the utilization of MetaMorph microscopy image analysis software (v7.8, Molecular Devices in Sunnyvale, CA, USA). The microscopic evaluation was performed independently by two observers in a blinded manner, with 20 randomly selected fields from each slide section examined at 400 × magnification. Any discrepancies in fibrosis quantification were resolved by consensus.

### 4.5. Immunohistochemistry

Mice kidney tissue was assessed for interstitial fibrosis and inflammation using immunohistochemical staining methods. Specifically, α-SMA (R&D Systems, Minneapolis, MN, USA) was employed to examine fibrotic changes, and the percentage of positively stained areas in relation to the total tissue area was quantified. Anti-F4/80 (Abcam, Cambridge, MA, USA) staining was utilized to detect macrophage infiltration as a marker of inflammation. F4/80-positive cells were counted manually in 20 randomly selected per high power fields (HPFs; ×400 magnification) per section in a blinded manner, and results are expressed as the number of positive cells per HPF. Additionally, the TUNEL assay was employed to assess apoptotic activity. TUNEL-positive cells were quantified as a percentage of total cells per HPF, assessed in a blinded manner across 20 randomly selected fields (×400 magnification) per section.

### 4.6. Western Blot

Fresh kidney tissues were lysed with radioimmunoprecipitation assay buffer (Thermo Fisher Scientific, Waltham, MA, USA) and the protein contents were measured using the Bradford assay (Bio-Rad, 500–0006). Protein concentrations were detected by the bicinchoninic acid assay protein assay kit (Pierce Biotechnology, Rockford, IL, USA). Equivalent amounts of each protein extract were heat denatured in 5 × sample buffer (2% sodium dodecyl sulfate, 62.5 mM Tris, pH 6.8, 0.01% bromophenol blue, 1.43 mM mercaptoethanol, and 0.1% glycerol), separated on 12% polyacrylamide gels, and electrophoretically transferred onto a polyvinylidene fluoride membrane (Millipore, Bedford, MA, USA). The membranes were first blocked with 5% skim milk for 1 h at room temperature, then incubated with primary antibody against collagen 1 (1:1000; Santa Cruz, Dallas, TX, USA), TGF-β (1:1000, Abcam, Cambridge, UK), α-SMA (1:10000; R&D Systems, Minneapolis, MN, USA), β-actin (1:10000; Sigma, St Louis, MO, USA), p-Smad3 and total Smad3 (1:1000; Cell Signaling Technology, Danvers, MA, USA), p-ERK 1/2 and total ERK1/2 (1:1000; Cell Signaling Technology, Danvers, MA, USA), and p-p38 and total p38 (1:1000; Cell Signaling Technology, Danvers, MA, USA) overnight at 4 °C, then incubated with corresponding secondary antibodies, including anti-mouse IgG-HRP (Cell signaling, Danvers, MA, USA), and anti-rabbit IgG-HRP (Cell signaling, Danvers, MA, USA) for 2 h at room temperature. Target proteins were visualized using Amersham ECL Western blotting Detection Reagent (GE Healthcare Life Science, IL, USA). Band densities were quantified using NIH ImageJ software (version 1.54, National Institutes of Health, Bethesda, MD, USA). All band intensities were normalized to β-actin as a loading control, and protein expression levels are presented as values relative to the control group, which was set to 1.0.

### 4.7. Real-Time Quantitative Reverse-Transcription Polymerase Chain Reaction

The mRNA expression levels of fresh frozen kidneys and HK-2 cells were analyzed by RT-qPCR. For RNA extraction, the TRIzol reagent (Invitrogen, Life Technologies, CA, USA) was utilized following the manufacturer’s protocol. Subsequently, reverse transcription to complimentary DNA (cDNA) was carried out using the High Capacity cDNA Reverse Transcription kit (Invitrogen, Life Technologies, CA, USA), and the cDNA was then amplified in the ABI 7500 sequence detection system (Applied Biosystems, Bedford, MA, USA) using the Power SYBR^®^ Green PCR Master Mix (Applied Biosystems, Bedford, MA, USA) with the following cycling conditions: 40 cycles of 95 °C for 5 s, 58 °C for 10 s, and 72 °C for 20 s. The sequences of primers were as follows: TGFβ-1 (forward: 5′- AAATCAACGGGATCAGCCCC-3′, reverse: 5′- CGCACACAGCAGTTCTTCTC-3′), collagen 1 (forward: 5′-GACATGTTCAGCTTTGTGGACCTC-3′, reverse: 5′-GGGACCCTTAGGCCATTGTGTA-3′), MCP-1 (forward: 5′-CACTCACCTGCTGCTACTCA-3′, reverse: 5′-GCTTGGTGACAAAAACTACAGC-3′), and glyceraldehyde-3-phosphate dehydrogenase (GAPDH) (forward: 5′-TGCATCCTGCACCACCAACT-3′, reverse: 5′-CCCGTTCAGCTCTGGGATGA-3′). The mRNA expression for each target gene was normalized relative to GAPDH expression. Relative quantification of gene expression was performed with the comparative CT method (ΔΔCT method).

### 4.8. Statistical Analysis

Data are expressed as mean ± standard deviation. Prior to statistical analysis, normality of data distribution was confirmed using the Shapiro–Wilk test, and homogeneity of variance was assessed using Levene’s test. The one-way ANOVA test with post hoc Tukey comparison was used for multiple group comparison between the control and treatment. Student’s *t*-test was further used to assess the difference between two groups. *p* < 0.05 was considered statistically significant, and all statistical analyses were performed using GraphPad Prism (version 8.0; GraphPad Software, San Diego, CA, USA).

## Data Availability

The original contributions presented in this study are included in the article. Further inquiries can be directed to the corresponding author.

## Supplementary Materials

The following supporting information can be downloaded at: https://www.mdpi.com/article/10.3390/ph19060960/s1; Table S1: LOX family expression changes after CsA administration; Table S2: Clinical characteristics changes after CsA and Compound #765 administration; Table S3: Histopathological changes after CsA and Compound #765 administration; Table S4: Changes in ECM proteins after CsA and Compound #765 administration; Table S5: Changes in immunohistochemical staining of F4/80 after CsA and Compound #765 administration; Table S6: Changes in immunohistochemical staining of TUNEL after CsA and Compound #765 administration; Table S7: Changes in proinflammatory cytokines after CsA and Compound #765 administration; Table S8: Changes in Smad3 and MAPK signaling activation after CsA and Compound #765 administration; Table S9: Changes in fibrotic gene expression in HK-2 cells after CsA and Compound #765 administration; Figure S1: Cell viability of HK-2 cells following treatment with Compound #765; Figure S2: Changes in fibrotic gene expression in HK-2 cells after CsA and Compound #765 administration.
