# Supplementary material for: Expression of Lysyl Oxidase-Related Protein and Effect of Lysyl Oxidase Inhibition in Cyclosporine-Induced Nephropathy Mouse Model
Source: Pharmaceuticals (Basel). 2026 Jun 21;19(6):960. doi: 10.3390/ph19060960 (PMC13306022; doi:10.3390/ph19060960)
Supplement: Supplementary file 1 [file pharmaceuticals-19-00960-s001.zip › pharmaceuticals-4296705-supplementary.pdf]

Supplemental Table S1. LOX family expression changes after CsA administration.

Gene	Mean ± SD	Mean Diff. (vs Control)	95% CI	P-value
Control	1.00 ± 0.11	−	−	
LOX	1.93 ± 0.36	−0.93	−1.68 to −0.18	0.010
LOXL1	1.77 ± 0.14	−0.77	−1.52 to −0.02	0.042
LOXL2	2.87 ± 0.55	−1.87	−2.62 to −1.12	<0.001
LOXL3	2.33 ± 0.19	−1.33	−2.08 to −0.57	<0.001
LOXL4	2.61 ± 0.32	−1.61	−2.36 to −0.86	<0.001

Data are expressed as mean ± SD (n = 4 per group). Mean difference and 95% confidence interval were calculated relative to the control group. P-values were derived from one-way ANOVA with Tukey's post hoc multiple comparisons test.

Abbreviations: CI, confidence interval; CsA, cyclosporine A; LOX, lysyl oxidase; LOXL, lysyl oxidase-like; SD, standard deviation.

Supplemental Table S2. Clinical characteristics changes after CsA and Compound #765 administration.

Parameter	Control	Compound #765	CsA	CsA + Compound #765	P value (Control vs CsA)	P value (CsA vs CsA+Compound#765)
Systolic BP	112.18 ± 2.82	114.11 ± 6.31	138.43 ± 6.12	123.22 ± 1.10	<0.001	0.008
Diastolic BP	57.54 ± 12.41	65.46 ± 24.46	113.91 ± 13.38	63.70 ± 15.75	0.008	0.017
Hemoglobin	11.43 ± 0.77	13.62 ± 0.70	6.55 ± 1.40	10.34 ± 0.40	<0.001	<0.001
BUN	17.73 ± 0.88	22.20 ± 1.43	33.25 ± 3.45	21.84 ± 2.95	<0.001	<0.001
Creatinine	0.11 ± 0.02	0.11 ± 0.02	0.30 ± 0.05	0.14 ± 0.03	<0.001	<0.001
UACR	13.78 ± 1.06	16.62 ± 0.98	44.15 ± 2.46	25.98 ± 3.25	<0.001	<0.001

Data are expressed as mean ± SD. P-values were derived from one-way ANOVA with Tukey's post hoc multiple comparisons test.

Abbreviations: BP, blood pressure; BUN, blood urea nitrogen; CsA, cyclosporine A; Hb, hemoglobin; SD, standard deviation; UACR, urine albumin-to-creatinine ratio.

Supplemental Table S3. Histopathological changes after CsA and Compound #765 administration.

Parameter	Control	Compound #765	CsA	CsA + Compound #765	P value (Control vs CsA)	P value (CsA vs CsA+Compound#765)
MT staining (positive pixels/mm²)	128.8 ± 5.0	142.2 ± 27.7	2491.8 ± 823.4	1266.1 ± 121.8	<0.001	<0.001
PSR staining (positive pixels/mm²)	234.2 ± 30.2	227.3 ± 32.3	2349.2 ± 191.6	1334.2 ± 349.5	<0.001	<0.001

Data are expressed as mean ± SD. P-values were derived from one-way ANOVA with Tukey's post hoc multiple comparisons test.

Abbreviations: CsA, cyclosporine A; MT, Masson's trichrome; PSR, picrosirius red; SD, standard deviation.

Supplemental Table S4. Changes in ECM proteins after CsA and Compound #765 administration.

Parameter	Control	Compound #765	CsA	CsA + Compound #765	P value (Control vs CsA)	P value (CsA vs CsA+Compound#765)
α-SMA IHC (positive pixels/mm²)	145.50 ± 27.08	193.94 ± 33.78	1945.15 ± 293.20	706.76 ± 31.27	<0.001	<0.001
α-SMA protein (α-SMA/Actin ratio)	1.00 ± 0.16	0.51 ± 0.17	3.41 ± 0.25	2.25 ± 0.18	<0.001	<0.001
Collagen 1 protein (Collagen 1/Actin ratio)	1.00 ± 0.05	0.78 ± 0.07	2.53 ± 0.20	1.11 ± 0.07	<0.001	<0.001
Collagen 1 mRNA (relative expression)	1.00 ± 0.22	0.92 ± 0.19	2.32 ± 0.35	1.35 ± 0.39	<0.001	0.004

Data are expressed as mean ± SD. P-values were derived from one-way ANOVA with Tukey's post hoc multiple comparisons test. α-SMA IHC data are expressed as absolute values (positive pixels/mm²). Protein and mRNA expression levels are presented as values relative to the control group, normalized to β-actin and GAPDH, respectively.

Abbreviations: α-SMA, alpha-smooth muscle actin; CsA, cyclosporine A; ECM, extracellular matrix; GAPDH, glyceraldehyde-3-phosphate dehydrogenase; IHC, immunohistochemistry; SD, standard deviation.

Supplemental Table S5. Changes in immunohistochemical staining of F4/80 after CsA and Compound #765 administration.

Parameter	Control	Compound #765	CsA	CsA + Compound #765	P value (Control vs CsA)	P value (CsA vs CsA+Compound#765)
F4/80 positive cells (cells/HPF)	2.00 ± 0.71	2.75 ± 0.83	21.25 ± 5.45	14.00 ± 1.58	<0.001	0.042

Data are expressed as mean ± SD. P-values were derived from one-way ANOVA with Tukey's post hoc multiple comparisons test.

Abbreviations: CsA, cyclosporine A; HPF, high power field; SD, standard deviation.

Supplemental Table S6. Changes in immunohistochemical staining of TUNEL after CsA and Compound #765 administration.

Parameter	Control	Compound #765	CsA	CsA + Compound #765	P value (Control vs CsA)	P value (CsA vs CsA+Compound#765)
TUNEL-positive cells (%)	0.25 ± 0.43	0.80 ± 0.75	74.75 ± 4.66	38.20 ± 2.14	<0.001	<0.001

Data are expressed as mean ± SD. P-values were derived from one-way ANOVA with Tukey's post hoc multiple comparisons test. TUNEL-positive cells are expressed as a percentage of total cells per high power field.

Abbreviations: CsA, cyclosporine A; HPF, high power field; SD, standard deviation; TUNEL, terminal deoxynucleotidyl transferase dUTP nick end labeling.

Supplemental Table S7. Changes in proinflammatory cytokines after CsA and Compound #765 LOXL2i administration.

Parameter	Control	Compound #765	CsA	CsA + Compound #765	P value (Control vs CsA)	P value (CsA vs CsA+Compound#765)
TGF-β1 protein (TGF-β1/Actin ratio)	1.00 ± 0.04	0.71 ± 0.12	1.70 ± 0.06	1.17 ± 0.14	<0.001	<0.001
TGF-β1 mRNA (TGF-β1/GAPDH)	1.00 ± 0.24	0.89 ± 0.23	2.15 ± 0.75	1.34 ± 0.34	0.022	0.107
MCP-1 mRNA (MCP-1/GAPDH)	1.00 ± 0.15	0.70 ± 0.18	4.35 ± 0.63	2.41 ± 0.39	<0.001	<0.001

Data are expressed as mean ± SD. P-values were derived from one-way ANOVA with Tukey's post hoc multiple comparisons test. Protein and mRNA expression levels are presented as values relative to the control group, normalized to β-actin and GAPDH, respectively.

Abbreviations: CsA, cyclosporine A; GAPDH, glyceraldehyde-3-phosphate dehydrogenase; MCP, monocyte chemoattractant protein; SD, standard deviation; TGF, transforming growth factor.

Supplementary Table S8. Changes in Smad3 and MAPK signaling activation after CsA and Compound #765 LOXL2i administration.

Parameter	Control	Compound #765	CsA	CsA + Compound #765	P value (Control vs CsA)	P value (CsA vs CsA+Compound#765)
p-Smad3/Smad3 ratio	1.00 ± 0.03	1.10 ± 0.07	2.42 ± 0.44	1.50 ± 0.15	0.001	0.019
p-p38/p38 ratio	1.00 ± 0.02	1.04 ± 0.02	2.38 ± 0.16	2.05 ± 0.08	<0.001	0.030
p-ERK1/2/ERK1/2 ratio	1.00 ± 0.12	1.03 ± 0.11	2.25 ± 0.28	1.21 ± 0.10	<0.001	0.001

Data are expressed as mean ± SD. P-values were derived from one-way ANOVA with Tukey's post hoc multiple comparisons test. All protein expression levels are presented as phosphorylated-to-total protein ratios relative to the control group.

Abbreviations: CsA, cyclosporine A; ERK, extracellular signal-regulated kinase; MAPK, mitogen-activated protein kinase; SD, standard deviation; Smad, small mothers against decapentaplegic.

Supplementary Table S9. Changes in fibrotic gene expression in HK-2 Cells after CsA and Compound #765 Administration.

Parameter	Control	Compound #765	CsA	CsA + Compound #765	P value (Control vs CsA)	P value (CsA vs CsA+Compound#765)
TGF-β1/GAPDH	1.00 ± 0.15	0.82 ± 0.07	2.51 ± 0.21	1.78 ± 0.20	<0.001	0.003
Collagen1/GAPDH	1.00 ± 0.11	0.84 ± 0.08	3.31 ± 0.14	2.26 ± 0.23	<0.001	<0.001
α-SMA/GAPDH	1.00 ± 0.14	0.88 ± 0.08	2.70 ± 0.23	1.80 ± 0.10	<0.001	<0.001

Note: Data are expressed as mean ± SD (n = 3 per group). Statistical comparisons were performed using one-way ANOVA with Tukey's post-hoc test.

Abbreviations: α-SMA, alpha-smooth muscle actin; CsA, cyclosporin A; GAPDH, glyceraldehyde-3-phosphate dehydrogenase; SD, standard deviation; TGF-β1, transforming growth factor-beta 1.

Supplementary Figure S1. Cell viability of HK-2 cells following treatment with Compound #765.

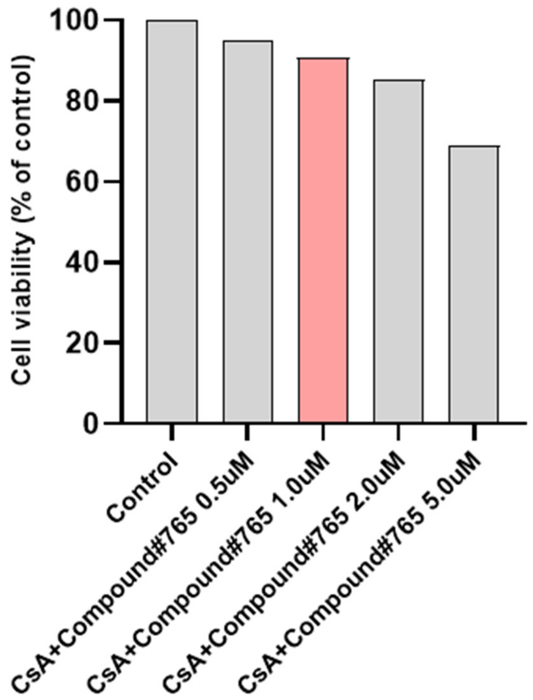

Note: Cell viability of HK-2 cells treated with increasing concentrations of Compound #765 (0.5, 1.0, 2.0, and 5.0 µM), expressed as a percentage of control. The pink bar indicates the selected working concentration (1.0 µM), at which cell viability remained above 90%. Data are expressed as mean ± SD (n = 3 per group).

Abbreviations: CsA, cyclosporin A; SD, standard deviation.

Supplementary Figure S2. Changes in fibrotic gene expression in HK-2 Cells after CsA and Compound #765 Administration.

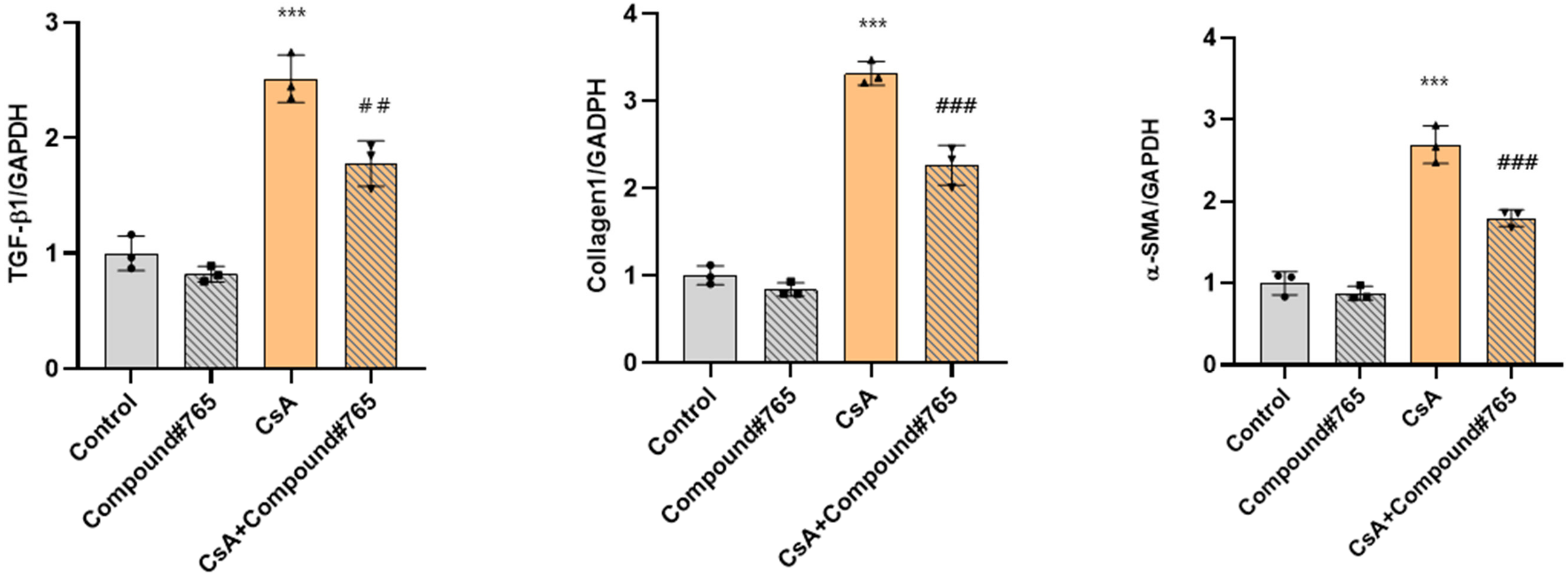

Note: mRNA expression levels of (a) TGF-β1, (b) Collagen type 1A, and (c) α-SMA in HK-2 cells treated with Control, Compound #765 (1 μM), CsA (5 μM), or CsA + Compound #765 (1 μM). mRNA expression was normalized to GAPDH. Data are expressed as mean ± SD (N = 3 per group). Statistical comparisons were performed using one-way ANOVA with Tukey's post-hoc test. vs. Control: ***P<0.001; vs. CsA: ##P<0.01, ###P<0.001.

Abbreviations: α-SMA, alpha-smooth muscle actin; CsA, cyclosporin A; GAPDH, glyceraldehyde-3-phosphate dehydrogenase; SD, standard deviation; TGF-β1, transforming growth factor-beta 1.
